# How Birth Season Affects Vulnerability to the Effect of Ambient Ozone Exposure on the Disease Burden of Hypertension in the Elderly Population in a Coastal City in South China

**DOI:** 10.3390/ijerph17030824

**Published:** 2020-01-28

**Authors:** Jing Huang, Tianfeng He, Guoxing Li, Xinbiao Guo

**Affiliations:** 1 Department of Occupational and Environmental Health Sciences, Peking University School of Public Health, Beijing 100191, China; jing_huang@bjmu.edu.cn (J.H.); guoxb@bjmu.edu.cn (X.G.); 2 Ningbo Municipal Center for Disease Control and Prevention, Ningbo 315010, China

**Keywords:** birth season, ozone, hypertension, disease burden, years of life lost

## Abstract

Birth season is an important factor that reflects prenatal nutritional conditions during early development, and which might have lifelong impacts on health. Moreover, ambient ozone pollution has been considered an important environmental risk factor for hypertension. However, whether birth season affects vulnerability to the effect of ambient ozone exposure on late-life hypertension is still unknown. A flexible case–crossover design was used to explore the effect of ambient ozone exposure on the disease burden of hypertension using years of life lost (YLL) in the elderly population in a coastal city in South China from 2013 to 2016. The influence of birth season was also explored. Ozone exposure was significantly associated with increased YLL from hypertension. The association was higher in the elderly individuals who were born in autumn than in those born in other seasons. Specifically, every 10 μg/m^3^ increase in ozone was associated with 0.68 (95% CI: 0.27, 1.10) YLL from hypertension in the elderly population born in autumn, while nonsignificant associations were found for those born in other seasons. The birth season, which affects the nutritional condition during early development, could affect vulnerability to the effect of ambient ozone exposure on the disease burden of hypertension in late life. The findings highlighted the importance of taking birth season into consideration when exploring the hypertensive effects of ozone exposure.

## 1. Introduction

Ambient air pollution has emerged as one of the most important environmental and public health concerns in the world [1]. As a key component of photochemical smog, ozone is a highly reactive and strongly oxidative secondary air pollutant [2]. With a rapid increase in the number of vehicles, the emission sources of air pollution, which previously mainly originated from conventional coal combustion, have gradually changed to a combination of coal combustion and motor vehicle emissions [3]. Thus, ozone pollution has increased substantially in recent years and has received increasing attention [4].

Hypertension is an important global health challenge worldwide considering its high prevalence [5]. Furthermore, along with rapid urbanization, economic development, and population aging, the prevalence of hypertension is increasing consistently in low- and middle-income countries [6]. As the major risk factor for cardiovascular diseases, hypertension has been identified as the leading risk factor for premature death and disability worldwide [7].

Among the many risk factors for hypertension, air pollution has been considered an important environmental influencing factor [8]. Nevertheless, studies on the associations between ambient ozone exposure and hypertension are far fewer than studies on particulate matter (PM), and the results have been inconsistent [9,10,11]. In addition, few studies have explored the effect of ozone exposure on the disease burden of hypertension using the years of life lost (YLL) indicator, which provides complementary information to that of mortality rate, because it takes the life expectancy at death into consideration [12] . Evidence of effect on elderly individuals, who may be more susceptible to the effects of ozone exposure, is even more scarce [13].

In addition, beyond the traditional demographic factors modifying the health effects of ozone exposure, such as age, gender, and marital status, whether birth season affects vulnerability to ozone exposure is unclear. Considering that birth season is an important index that reflects the prenatal nutritional condition during early development, which will exert lifelong impacts on health [14], it is interesting to explore the influence of this factor on the possible hypertensive effects of ozone exposure.

Therefore, the aim of this study is to explore the associations between ambient ozone exposure and the disease burden of hypertension using the indicator of YLL in the elderly. Furthermore, the influence of birth season was investigated for the first time. The results will provide important evidence for ozone pollution control and susceptible population protection.

## 2. Materials and Methods 

### 2.1. Study Site and Data Collection

Our study site is Ningbo city, which is located in the Zhejiang Province in China. It is the world’s fourth-largest port city and an important commercial and financial center in South China. The total population of Ningbo is 5.83 million, and 6.27% of individuals are ≥75 years old (http://vod.ningbo.gov.cn:88/nbtjj/tjnj/2015nbnj/indexch.htm).

Daily 8-hour maximum concentrations of ozone and daily concentrations of fine particles (PM_2.5_), nitrogen dioxide (NO_2_), and sulfur dioxide (SO_2_) were collected from the Environment Monitoring Center of Ningbo between 1 January 2013 and 31 December 2016. The air pollutant levels were monitored at 8 monitoring sites covering urban and suburban areas in Ningbo, and the monitoring methods were performed according to the Chinese National Ambient Air Quality Standards. The averaged data from these sites reflected the air pollution levels of the whole city. Daily meteorological data, including temperature and relative humidity, were obtained from the Ningbo Meteorological Bureau. The proportion of missing data was less than 1.0%, and the missing values were substituted with the daily mean value.

Daily mortality data on death in the elderly population were obtained from the Ningbo Municipal Center for Disease Control and Prevention. The elderly subjects who were retained for the calculation of the YLL were the ones whose primary cause of death was hypertension. The diagnose of death caused by hypertension used the same method as in a previous study in 272 Chinese cities, “according to the International Classification of Diseases, 10th revision, daily mortality counts were further categorized into hypertension (codes: I10–I15)” [9].

Elderly individuals were those who were ≥75 years old according to previous studies [15,16]. All data were restricted to registered residents only, and the dataset comprised information on gender, age, birth date, and other factors. Thus, deaths from hypertension could be stratified by birth season according to the birth date of the individual. Based on the definition of seasons of the China Meteorological Administration, spring ranges from March to May, summer from June to August, autumn from September to November, and winter from December to February. 

We calculated daily YLL from hypertension in the elderly population by matching each patient’s age to the World Health Organization (WHO) standard life table (Supplemental Material, Table S1). Daily YLL was calculated by summing the YLL of all the elderly individuals who died from hypertension on the same day. Furthermore, the daily YLL was stratified by birth season.

### 2.2. Statistical Analysis

The Spearman correlation function was used to analyze the correlations between ozone and other air pollutants, as well as the meteorological factors. Time-stratified case–crossover using conditional logistic regression was adopted to explore the effects of ozone exposure on YLL from hypertension. This design is a special case of time series analysis. We used a Poisson regression model that combines the case–crossover design with penalized splines [17].

The model is as follows:

$$YLL_{t}=\alpha +\sum_{i=1}^{q}\beta i\left(\mathrm{Xi}\right)+\sum_{j=1}^{p}\mathrm{fj}\left(\mathrm{Cj},\mathrm{df}\right)+\mathrm{Wt}\left(\mathrm{week}\right)\;+\;\mathrm{Strata}$$

In the model, *YLL_t_* is the observed daily YLL from hypertension at day *t*; *α* is the intercept; *β* is the coefficient of YLL associated with a unit increase in ozone; *Xi* is the daily mean concentration of ozone; *Cj* represents the confounding factors, including daily temperature and daily relative humidity; *f_j_* is the smooth function (p spline); and *W_t_*(week) is the dummy variable for day of week on day *t*. Strata is a categorical variable of the year and calendar month used to control for long-term trends. Degrees of freedom for daily temperature and relative humidity were set to 3 according to previous studies [16,18]. Considering the lag effects of temperature, the 14-day moving average of temperature was used [19]. For relative humidity, the average value of the current day was used in the models.

To investigate the lag effects of ozone exposure, potentially delayed and cumulative associations were estimated. The delayed effects were estimated by using a single day lag (from lag0 to lag7), and then the cumulative associations were estimated using the moving average over the lag periods from moving average concentrations of day 0 and day 1 (mv01) to moving average concentrations of day 0 to day 7 (mv07). The results are shown as changes in daily YLL from hypertension per 10 μg/m^3^ increase in ozone on different lag days.

To visualize the possible nonlinear relationship between ozone exposure and YLL from hypertension, a penalized spline smoothing function was used to plot the exposure–response curves. In addition, the analyses were stratified by birth season, and the differences between birth seasons were analyzed by subgroup comparisons, as shown below.

$$\left(\beta_{1}\;-\;\beta_{2}\right)\;\pm \;1.96\sqrt{SE_{1}^{2}+SE_{2}^{2}}$$

where *β_1_* and *β_2_* are the estimates for the two subgroups (e.g., autumn and spring) and *SE_1_* and *SE_2_* are their respective standard errors.

A single-pollutant model was used to examine the main associations of ozone exposure with daily YLL from hypertension, while two-pollutant models with PM_2.5_, NO_2_, or SO_2_ added were used to examine the robustness of the associations.

Furthermore, we evaluated the impacts of ozone exposure on hypertension mortality. The independent variable, lag structure, and relevant degrees of freedom in the model were similar to those in the YLL models, expect the time series function with the Poisson link under a case crossover framework was used considering the daily death counts following a Poisson distribution. The results are presented as changes in the excess risk of death from hypertension associated with each 10 μg/m^3^ increase in daily 8-hour maximum ozone on different lag days.

The study was approved by the Institutional Review Board of Ningbo Municipal Center for Disease Control and Prevention (No. IRB 201603). R software was used for all analyses (Version 3.1.2, http://www.R-project.org/). Statistical significance was defined as two-sided *p* < 0.05.

## 3. Results

### 3.1. Description

**Figure 1:**
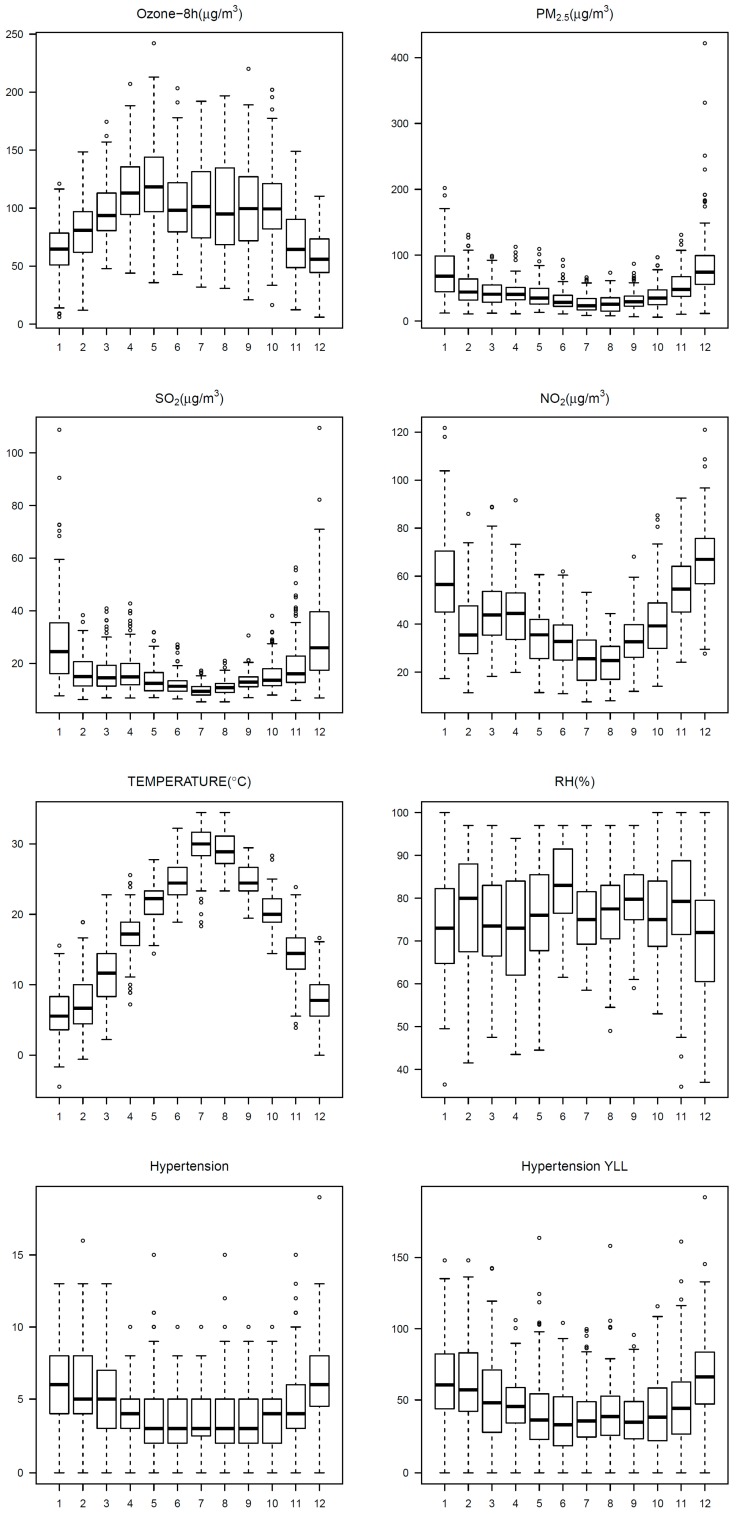
Box plots of monthly air pollutant concentrations, meteorological conditions, number of deaths, and the corresponding years of life lost (YLL) from hypertension in Ningbo, China, from 2013 to 2016.

The mean daily 8-hour maximum ozone concentration was 93.3 μg/m^3^ during from 2013 to 2016 in Ningbo, China (Table 1). The concentrations of ozone were relatively higher in summer months, while the concentrations of PM_2.5_, NO_2_, and SO_2_ were higher in winter months (Figure 1). Correlations between ozone and other air pollutants, as well as the meteorological factors, are presented in Table S2 in the Supplementary Materials. Ozone was negatively associated with PM_2.5_, NO_2_, and SO_2_. 

From 2013 to 2016, a total of 6525 deaths from hypertension in the elderly population were recorded, and the corresponding YLL from hypertension was 70,014 years. The daily number of deaths and the corresponding YLL from hypertension in the elderly individuals born in different seasons are shown in Table 2. Relatively higher daily YLL from hypertension was found in those born in autumn and winter. Daily YLL and death number in males and females are also presented. 

### 3.2. Associations between Ambient Ozone and YLL from Hypertension

**Figure 2:**
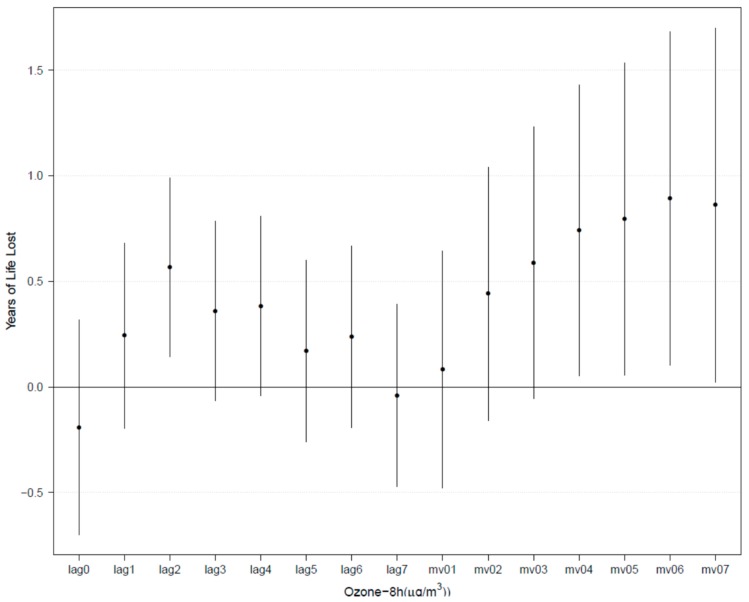
Estimated changes with 95% confidence intervals in years of life lost (YLL) from hypertension associated with each 10 μg/m^3^ increase in ozone on different lag days in the elderly population in Ningbo, China, from 2013 to 2016.

Estimated changes in YLL from hypertension associated with increments in daily 8-hour maximum ozone on different lag days in the elderly population are presented in Figure 2. Generally, the effects showed a positive trend and peaked at 6-day moving average concentration (mv06), with each 10 μg/m^3^ increase in daily 8-hour maximum ozone associated with an increase in YLL from hypertension in the elderly population of 0.89 years (95% CI: 0.10, 1.68). Because the effects were strongest for mv06, we used this moving average in our main analysis.

### 3.3. Modification of Birth Season

When the analysis was stratified by birth season, YLL from hypertension related to ozone exposure was significantly higher among the autumn-born elderly individuals than among those born in other seasons. Specifically, each 10 μg/m^3^ increase in daily 8-hour maximum ozone corresponded to an increase in YLL from hypertension of 0.68 years (95% CI: 0.27, 1.10) in those born in autumn, while nonsignificant associations were found for other birth seasons.

In two-pollutant models, the estimates of the associations with ozone in different birth seasons did not change much when PM_2.5_, NO_2_, or SO_2_ was added. For instance, when PM_2.5_ was added to the model, a 10 μg/m^3^ increase in daily 8-hour maximum ozone was associated with an increase in YLL from hypertension of 0.74 years (95% CI: 0.29, 1.19), which was significantly higher than for those born in other seasons. Nonsignificant differences were found in YLL from hypertension per increase in exposition to ozone between males and females (Table 3).

The excess risk of hypertension mortality associated with ozone exposure showed the same trend as the results for YLL. The excess risk was highest in those who were born in autumn. Nevertheless, significant differences were only found for autumn-born and winter-born individuals (Supplemental Material, Table S3). The results indicated that YLL may be more sensitive than mortality when exploring the health effects of air pollutants.

### 3.4. Exposure–Response Relationship

**Figure 3:**
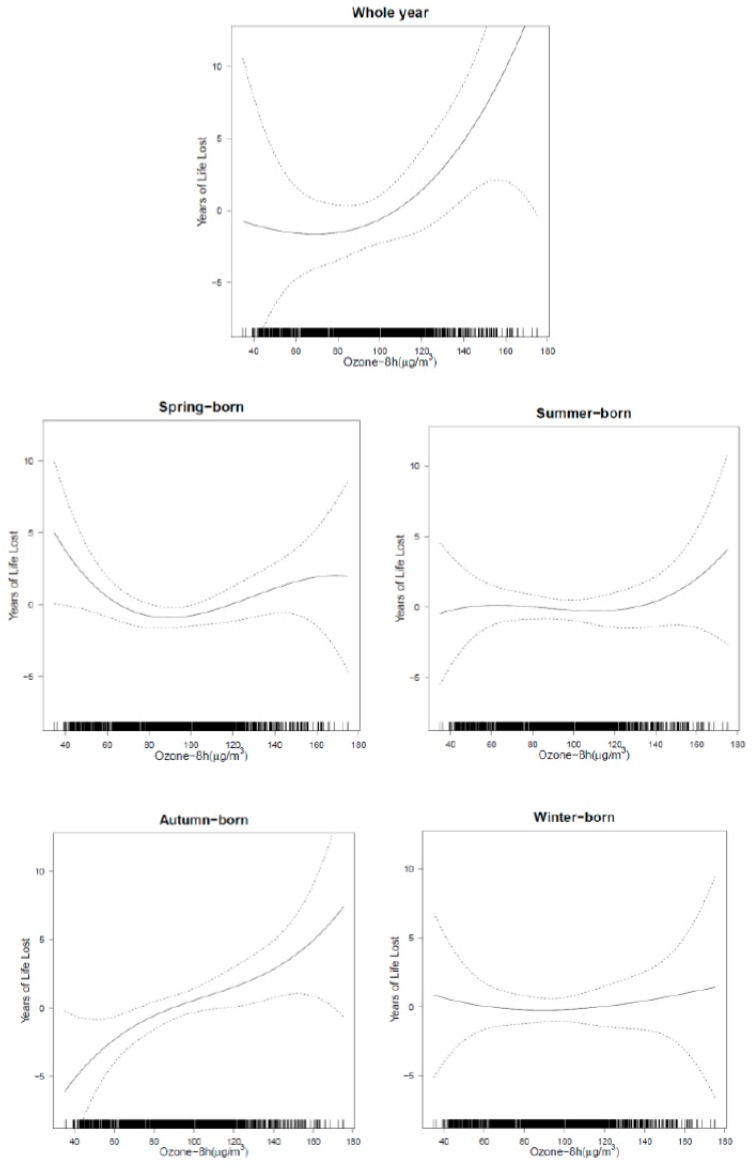
Exposure–response curves of ozone and years of life lost from hypertension in the elderly individuals born in different seasons in Ningbo, China, from 2013 to 2016.

The exposure–response curves of ozone exposure and YLL from hypertension in the elderly individuals born in four seasons showed different patterns. An approximately linear relationship was shown for ozone exposure in those born in autumn and the curve was relatively steep, while the curves for other birth seasons were flatter (Figure 3).

The exposure–response curves were based on the results of mv06. The upper and lower dotted lines indicate 95% confidence intervals.

## 4. Discussion

In this study, a flexible case–crossover design was used to investigate the associations between ambient ozone exposure and the disease burden of hypertension in elderly individuals born in different birth seasons. We found that ambient ozone exposure was significantly associated with YLL from hypertension in the elderly population. Furthermore, it was very interesting to find that the increased burden of hypertension related to ozone exposure was significantly higher in the individuals who were born in autumn than in those born in other seasons.

High ozone concentrations in urban and industrial regions worldwide have been a major air quality issue [20]. With the rapid increase in fossil fuel consumption in China over the past decades, the emission of photochemical precursors of ozone has increased sharply, raising concerns about worsening ozone pollution [21]. Our study site was in the region of the Yangtze River Delta of South China, where increased ambient ozone concentration has been reported in recent years [4]. We found that the daily mean concentration of ambient ozone in this study was approximately equal to the WHO air quality guideline limit for ozone, and was much higher than the ozone concentrations reported in other regions [2,22]. 

However, less is known about the health effects of ozone pollution compared with PM [23]. Previous findings on the relationship between ambient ozone exposure and hypertension have been contradictory. A systematic review and meta-analysis indicated that short-term exposure to ozone had a positive relationship with hypertension but lacked statistical significance [11]. Furthermore, most of the studies focus on hypertension mortality [9]. Our results add to the current literature by examining YLL, which is a better measurement of disease burden that takes both number of deaths and life expectancy into consideration. We found that ozone was significantly associated with YLL from hypertension in the elderly in Ningbo, Zhejing province, China. 

The mechanisms underlying the hypertensive effects of ambient ozone exposure are somewhat biologically plausible. Previous studies have found that ozone exposure contributed to an increased risk of hypertension, and the contributing factors might include circulating biomarkers of inflammation, oxidative stress, coagulation, platelet activation, and metabolomic change, which may cause increased sympathetic tone and vascular endothelial dysfunction [24,25,26]. However, the exact mechanisms of the hypertensive effects of ozone exposure remain to be elucidated in further investigations.

Relatively stronger hypertensive effects of ambient ozone exposure in the elderly population were found in this study. Specifically, each 10 μg/m^3^ increase in daily 8-hour maximum ozone concentration was associated with 2.01% (95% CI: 0.35%, 3.67%) higher hypertension mortality in the elderly population (Supplemental Material, Table S3). A study conducted in 272 Chinese cities found that each 10 μg/m^3^ increase in daily 8-hour maximum ozone concentration was associated with 0.66% (95% CI: 0.02%, 1.30%) higher daily mortality from hypertension in South China in the general population [9]. This may have been because elderly individuals are more vulnerable to the effect of ozone exposure, considering that many of them may have comorbidity of cardiovascular diseases, and their physical condition, immunity, and self-repairing ability are weaker than those of young people [13].

An increasing trend of hypertension prevalence has been reported. Based on a national survey in China between 2012 and 2015, the prevalence of hypertension among the Chinese adult population ≥ 18 years of age had already reached 23.2% [27]. Furthermore, hypertension became the leading risk factor contributing to disease burden in China in 2017 [28]. Thus, considering the ubiquitous existence of ambient ozone pollution and the substantial health risk of hypertension, more efforts should be taken to reduce the disease burden of hypertension associated with ambient ozone exposure, especially in the elderly population.

Furthermore, we found some interesting evidence of the influence of birth season on the associations between ozone exposure and the disease burden of hypertension in the elderly population. The individuals who were born in autumn had significantly greater YLL from hypertension than those born in other seasons. The results were also relatively robust after adjusting for copollutants, including PM_2.5_, NO_2_, and SO_2_.

According to the Development Origins of Health and Disease (DOHaD), environmental factors in the early stages of human life affect susceptibility to noncommunicable diseases in adults [29]. Recent research has highlighted the role of early-life factors that affect late-life mortality [30]. In particular, adult mortality was affected by nutritional status during the prenatal period, although controversy still exists regarding these results [14].

Hypertension is a major risk factor for cardiovascular diseases, which are recognized as having early origins through altered development programming due to adverse environmental conditions during development [31]. Birth season is an important indicator that reflects the nutritional condition during pregnancy [14], which may exert lifelong impacts on disease risk [32]. Thus, seasonal differences in nutritional condition in early life may explain the vulnerability to the hypertensive effects of ozone exposure in late life. Those who were born in autumn had a higher disease burden of hypertension in our study. This finding may be explained by the fact that the first trimester of pregnancy, which is also the key conceptional period, occurred during the winter, when fresh fruits, vegetables, and protein supply were insufficient. This could possibly lead to undernutrition in utero, especially during famine time, influencing the offspring’s vulnerability to hypertension in late life.

Programming of metabolism in very early life by undernutrition could lead to increased lifelong vulnerability to risk factors for cardiometabolic disorders [33]. It was proposed that poor early nutrition, especially undernutrition in utero, was at least partly associated with cardiovascular diseases in an adult population [34]. On the one hand, fetal undernutrition may make an infant vulnerable to postnatal stress; on the other hand, this may mean that the mother’s metabolic capacity cannot function well, possibly because of undernutrition during infancy [35]. Furthermore, an experimental animal study confirmed that it is possible to program high blood pressure, which is a marked feature of hypertension, by manipulating the nutritional status of the mother during pregnancy [36].

Thus, the results of our study suggest that it is important to explore the possible modification effects of birth season, which reflects the prenatal nutrition condition during early development, on the relationship between ozone exposure and the disease burden of hypertension in late life. 

Our study has several strengths. First, the evidence of the effects of ambient ozone on hypertension is limited, especially in the elderly population. Our study added results to the current literature on this important issue. Second, to our knowledge, this is the first study to explore the impacts of birth season on the associations between ozone and the disease burden of hypertension. This work contributes to the understanding of the influence of birth season and indicates that the prenatal nutritional condition during early life should be considered when exploring the late life mortality associated with air pollution. Third, the indicator of YLL was used in our study, which is more informative for disease burden assessment than mortality, and provided evidence for ozone pollution control and resource allocation.

However, some limitations should be noted in our study. First, exposure measurement errors existed because ambient air pollution data were collected from fixed monitoring stations as a proxy for personal exposure, which may have led to greater heterogeneity of the effects. Second, as an ecological study, individual factors such as gender, lifestyle factors, time spent outdoors or indoors, use of air conditioning, and medication were not controlled in the study due to data availability. Third, the study was performed in a single city; thus, caution should be taken when generalizing the results to other regions, and more confirmation studies need to be performed in the future.

## 5. Conclusions

This study showed the hypertensive effects of ambient ozone exposure in the elderly population and provided new insight into the ways in which birth season may influence the related associations. Those who were born in autumn had the highest risk compared with those who were born in other seasons. Considering that birth season is an important indicator that reflects prenatal nutrition condition during early development, which may exert lifelong impacts on disease risk, the results suggested that this demographic factor should be considered when exploring the health effects of ozone exposure. The findings will be informative for policy making regarding ambient ozone pollution control and vulnerable population protection.

## Figures and Tables

**Table 1:** Daily air pollutant concentrations and meteorological conditions in Ningbo, China, from 2013 to 2016.

Variables	Mean ± SD	Minimum	P25	Median	P75	Maximum
**Air pollutants**						
Ozone-8h (μg/m^3^)	93.3 ± 37.9	6.0	65.0	90.0	117.0	242.1
PM_2.5_ (μg/m^3^)	45.5 ± 32.0	5.9	25.6	37.6	56.0	421.7
NO_2_ (μg/m^3^)	41.6 ± 18.3	7.6	28.1	38.6	52.5	121.8
SO_2_ (μg/m^3^)	16.9 ± 10.9	5.4	10.4	13.5	19.1	109.5
**Meteorological conditions**						
Temperature (°C)	18.0 ± 8.7	−4.4	10.0	18.9	25.0	34.4
Relative humidity (%)	76.2 ± 11.8	36.0	68.5	76.0	85.0	100.0

Note: P25 indicates the 25th percentile, P75 indicates the 75th percentile.

**Table 2:** Daily number of deaths and years of life lost from hypertension in the elderly individuals born in different seasons in Ningbo, China, from 2013 to 2016.

Variables	Mean ± SD	Minimum	P25	Median	P75	Maximum
**Daily death counts**						
Whole year	4.5 ± 2.6	0.0	3.0	4.0	6.0	19.0
Male	1.9 ± 1.5	0.0	1.0	2.0	3.0	8.0
Female	2.6 ± 1.8	0.0	1.0	2.0	4.0	12.0
Spring-born	0.9 ± 1.0	0.0	0.0	1.0	1.0	6.0
Summer-born	0.9 ± 1.0	0.0	0.0	1.0	1.0	5.0
Autumn-born	1.3 ± 1.2	0.0	0.0	1.0	2.0	8.0
Winter-born	1.4 ± 1.2	0.0	0.0	1.0	2.0	8.0
**Daily years of life lost (years)**						
Whole year	47.9 ± 28.2	0.0	27.3	44.1	64.2	192.3
Male	21.0 ± 17.4	0.0	8.8	17.8	31.0	92.8
Female	26.9 ± 18.9	0.0	13.3	24.4	37.1	130.7
Spring-born	9.2 ± 11.1	0.0	0.0	6.6	14.4	70.8
Summer-born	9.9 ± 11.3	0.0	0.0	8.2	15.2	69.1
Autumn-born	14.3 ± 13.9	0.0	0.0	12.1	21.8	87.4
Winter-born	14.5 ± 13.6	0.0	0.0	12.1	22.6	88.6

**Table 3:** Changes in years of life lost (YLL) from hypertension per 10 μg/m^3^ increase in ozone in the elderly individuals born in different seasons using single and two-pollutant models in Ningbo, China, from 2013 to 2016.

Variables	All (95% CI)	Spring-born (95% CI)	Summer-born (95% CI)	Autumn-born (95% CI)	Winter-born (95% CI)
**Single-pollutant model**	0.89(0.10, 1.68)	0.06(−0.28, 0.40) *	0.10(−0.24, 0.46) *	0.68(0.27, 1.10)	0.04(−0.37, 0.46) *
Male	0.43(−0.07, 0.95)	0.08(−0.13, 0.30)	0.00(−0.25, 0.24)	0.27(0.00, 0.55)	0.09 (−0.20,0.37)
Female	0.46(−0.10, 1.01)	−0.01(−0.26, 0.25)	0.11(−0.15, 0.36)	0.41(0.10, 0.71)	−0.04 (−0.33,0.25)
**Two-pollutant model**					
+PM_2.5_	1.03(0.16, 1.90)	0.12(−0.26, 0.50) *	0.15(−0.24, 0.54) *	0.74(0.29, 1.19)	0.02(−0.44, 0.48) *
+NO_2_	0.98(0.16, 1.80)	0.08(−0.28, 0.44) *	0.12(−0.25, 0.49) *	0.74(0.31, 1.17)	0.04(−0.40, 0.47) *
+SO_2_	0.98(0.16, 1.79)	0.03(−0.33, 0.39) *	0.11(−0.26, 0.48) *	0.77(0.34, 1.19)	0.07(−0.36, 0.50) *

Changes are presented at mv06. The unit of the values is years. Autumn is set as the reference season. Note: *p < 0.05 compared with the changes in years of life lost in the autumn-born older population.

## Supplementary Materials

The following are available online at https://www.mdpi.com/1660-4601/17/3/824/s1. Table S1: WHO standard life table for years of life lost. Table S2: Spearman correlations between air pollutants and meteorological conditions in Ningbo, China, 2013–2016. Table S3: Changes in excess risk of hypertension mortality associated with 10 μg/m^3^ increase in ozone exposure in the elderly in Ningbo, China, 2013–2016.

## References

[1] Cohen A.J., Brauer M., Burnett R., Anderson H.R., Frostad J., Estep K., Balakrishnan K., Brunekreef B., Dandona L., Dandona R. (2017). Estimates and 25-year trends of the global burden of disease attributable to ambient air pollution: An analysis of data from the Global Burden of Diseases Study 2015. Lancet.

[2] Bell M.L., McDermott A., Zeger S.L., Samet J.M., Dominici F. (2004). Ozone and short-term mortality in 95 US urban communities, 1987–2000. JAMA.

[3] Kan H., Chen R., Tong S. (2012). Ambient air pollution, climate change, and population health in China. Environ. Int..

[4] Huang J., Pan X., Guo X., Li G. (2018). Health impact of China’s Air Pollution Prevention and Control Action Plan: An analysis of national air quality monitoring and mortality data. Lancet Planet Health.

[5] Kearney P.M., Whelton M., Reynolds K., Muntner P., Whelton P.K., He J. (2005). Global burden of hypertension: Analysis of worldwide data. Lancet.

[6] Mills K.T., Bundy J.D., Kelly T.N., Reed J.E., Kearney P.M., Reynolds K., Chen J., He J. (2016). Global Disparities of Hypertension Prevalence and Control: A Systematic Analysis of Population-Based Studies From 90 Countries. Circulation.

[7] GBD 2017 Risk Factor Collaborators (2018). Global, regional, and national comparative risk assessment of 84 behavioural, environmental and occupational, and metabolic risks or clusters of risks for 195 countries and territories, 1990-2017: A systematic analysis for the Global Burden of Disease Study 2017. Lancet.

[8] Yang B.Y., Qian Z.M., Vaughn M.G., Nelson E.J., Dharmage S.C., Heinrich J., Lin S., Lawrence W.R., Ma H., Chen D.H. (2017). Is prehypertension more strongly associated with long-term ambient air pollution exposure than hypertension? Findings from the 33 Communities Chinese Health Study. Environ. Pollut..

[9] Yin P., Chen R., Wang L., Meng X., Liu C., Niu Y., Lin Z., Liu Y., Liu J., Qi J. (2017). Ambient Ozone Pollution and Daily Mortality: A Nationwide Study in 272 Chinese Cities. Environ. Health Perspect..

[10] Yang B.Y., Qian Z., Howard S.W., Vaughn M.G., Fan S.J., Liu K.K., Dong G.H. (2018). Global association between ambient air pollution and blood pressure: A systematic review and meta-analysis. Environ. Pollut..

[11] Cai Y.Y., Zhang B., Ke W.X., Feng B.X., Lin H.L., Xiao J.P., Zeng W.L., Li X., Tao J., Yang Z.Y. (2016). Associations of Short-Term and Long-Term Exposure to Ambient Air Pollutants With Hypertension: A Systematic Review and Meta-Analysis. Hypertension.

[12] Zeng Q., Ni Y., Jiang G., Li G., Pan X. (2017). The short term burden of ambient particulate matters on non-accidental mortality and years of life lost: A ten-year multi-district study in Tianjin, China. Environ. Pollut..

[13] Zhang J., Chen Q., Wang Q., Ding Z., Sun H., Xu Y. (2019). The acute health effects of ozone and PM_2.5_ on daily cardiovascular disease mortality: A multi-center time series study in China. Ecotoxicol. Environ. Saf..

[14] Doblhammer G., Vaupel J.W. (2001). Lifespan depends on month of birth. Proc. Natl. Acad. Sci. USA.

[15] Lu F., Zhou L., Xu Y., Zheng T., Guo Y., Wellenius G.A., Bassig B.A., Chen X., Wang H., Zheng X. (2015). Short-term effects of air pollution on daily mortality and years of life lost in Nanjing, China. Sci. Total Environ..

[16] Yang J., Ou C.Q., Song Y.F., Li L., Chen P.Y., Liu Q.Y. (2016). Estimating years of life lost from cardiovascular mortality related to air pollution in Guangzhou, China. Sci. Total Environ..

[17] Guo Y., Barnett A.G., Pan X., Yu W., Tong S. (2011). The Impact of Temperature on Mortality in Tianjin, China: A Case-crossover Design with A Distributed Lag Non-linear Model. Environ. Health Perspect..

[18] Huang J., Li G., Qian X., Xu G., Zhao Y., Huang J., Liu Q., He T., Guo X. (2018). The burden of ischemic heart disease related to ambient air pollution exposure in a coastal city in South China. Environ. Res..

[19] Chen R., Cai J., Meng X., Kim H., Honda Y., Guo Y.L., Samoli E., Yang X., Kan H. (2014). Ozone and daily mortality rate in 21 cities of East Asia: How does season modify the association?. Am. J. Epidemiol..

[20] Monks P.S., Archibald A.T., Colette A., Cooper O., Coyle M., Derwent R., Fowler D., Granier C., Law K.S., Mills G.E. (2015). Tropospheric ozone and its precursors from the urban to the global scale from air quality to short-lived climate forcer. Atmos. Chem. Phys..

[21] Wang T., Xue L., Brimblecombe P., Lam Y.F., Li L., Zhang L. (2017). Ozone pollution in China: A review of concentrations, meteorological influences, chemical precursors, and effects. Sci. Total Environ..

[22] Huang W.H., Chen B.Y., Kim H., Honda Y., Guo Y.L. (2019). Significant effects of exposure to relatively low level ozone on daily mortality in 17 cities from three Eastern Asian Countries. Environ. Res..

[23] Li G., Xue M., Zeng Q., Cai Y., Pan X., Meng Q. (2017). Association between fine ambient particulate matter and daily total mortality: An analysis from 160 communities of China. Sci. Total Environ..

[24] Goodman J.E., Prueitt R.L., Sax S.N., Pizzurro D.M., Lynch H.N., Zu K., Venditti F.J. (2015). Ozone exposure and systemic biomarkers: Evaluation of evidence for adverse cardiovascular health impacts. Crit. Rev. Toxicol..

[25] Day D.B., Xiang J., Mo J., Li F., Chung M., Gong J., Weschler C.J., Ohman-Strickland P.A., Sundell J., Weng W. (2017). Association of Ozone Exposure With Cardiorespiratory Pathophysiologic Mechanisms in Healthy Adults. JAMA. Intern. Med..

[26] Xia Y., Niu Y., Cai J., Lin Z., Liu C., Li H., Chen C., Song W., Zhao Z., Chen R. (2018). Effects of Personal Short-Term Exposure to Ambient Ozone on Blood Pressure and Vascular Endothelial Function: A Mechanistic Study Based on DNA Methylation and Metabolomics. Environ. Sci. Technol..

[27] Wang Z., Chen Z., Zhang L., Wang X., Hao G., Zhang Z., Shao L., Tian Y., Dong Y., Zheng C. (2018). Status of Hypertension in China: Results From the China Hypertension Survey, 2012–2015. Circulation.

[28] Zhou M., Wang H., Zeng X., Yin P., Zhu J., Chen W., Li X., Wang L., Wang L., Liu Y. (2019). Mortality, morbidity, and risk factors in China and its provinces, 1990–2017: A systematic analysis for the Global Burden of Disease Study 2017. Lancet.

[29] Gluckman P.D., Hanson M.A., Beedle A.S. (2007). Early life events and their consequences for later disease: A life history and evolutionary perspective. Am. J. Hum. Biol..

[30] Wilding S., Ziauddeen N., Smith D., Roderick P., Alwan N.A. (2019). Maternal and early-life area-level characteristics and childhood adiposity: A systematic review. Obes. Rev..

[31] Chehade H., Simeoni U., Guignard J.P., Boubred F. (2018). Preterm Birth: Long Term Cardiovascular and Renal Consequences. Curr. Pediatr. Rev..

[32] Barker D.J., Gluckman P.D., Godfrey K.M., Harding J.E., Owens J.A., Robinson J.S. (1993). Fetal nutrition and cardiovascular disease in adult life. Lancet.

[33] Whalley L.J., Dick F.D., McNeill G. (2006). A life-course approach to the aetiology of late-onset dementias. Lancet Neurol..

[34] Barker D.J. (2007). The origins of the developmental origins theory. J. Intern. Med..

[35] Barker D.J., Osmond C., Forsen T.J., Kajantie E., Eriksson J.G. (2007). Maternal and social origins of hypertension. Hypertension.

[36] Duque-Guimaraes D.E., Ozanne S.E. (2013). Nutritional programming of insulin resistance: Causes and consequences. Trends Endocrinol. Metab..

