# Supplementary material for: How Birth Season Affects Vulnerability to the Effect of Ambient Ozone Exposure on the Disease Burden of Hypertension in the Elderly Population in a Coastal City in South China
Source: Int J Environ Res Public Health. 2020 Jan 28;17(3):824. doi: 10.3390/ijerph17030824 (PMC7036818; doi:10.3390/ijerph17030824)
Supplement: Supplementary file 1 [file ijerph-17-00824-s001.pdf]

International Journal of
Environmental Research and Public Health

MDPI

Supplemental material

How Birth Season Affects Vulnerability to the Effect of Ambient Ozone Exposure on the Disease Burden of Hypertension in the Elderly Population in a Coastal City in South China

Table S1. WHO standard life table for years of life lost.

Table S2. Spearman correlations between air pollutants and meteorological conditions in Ningbo, China, 2013–2016.

Table S3. Changes in excess risk of hypertension mortality associated with 10 μg/m^3 increase in ozone exposure in the elderly in Ningbo, China, 2013–2016.

Table S1. World Health Organization (WHO) standard life table for years of life lost.

Age	SEYLL	Age	SEYLL	Age	SEYLL
0	91.94	35	57.15	70	23.15
1	91	36	56.16	71	22.23
2	90.01	37	55.17	72	21.31
3	89.01	38	54.18	73	20.4
4	88.02	39	53.19	74	19.51
5	87.02	40	52.2	75	18.62
6	86.02	41	51.21	76	17.75
7	85.02	42	50.22	77	16.89
8	84.02	43	49.24	78	16.05
9	83.03	44	48.25	79	15.22
10	82.03	45	47.27	80	14.41
11	81.03	46	46.28	81	13.63
12	80.03	47	45.3	82	12.86
13	79.03	48	44.32	83	12.11
14	78.04	49	43.34	84	11.39
15	77.04	50	42.36	85	10.7
16	76.04	51	41.38	86	10.03
17	75.04	52	40.41	87	9.38
18	74.05	53	39.43	88	8.76
19	73.05	54	38.46	89	8.16
20	72.06	55	37.49	90	7.6
21	71.06	56	36.52	91	7.06
22	70.07	57	35.55	92	6.55
23	69.07	58	34.58	93	6.07
24	68.08	59	33.62	94	5.6
25	67.08	60	32.65	95	5.13
26	66.09	61	31.69	96	4.65
27	65.09	62	30.73	97	4.18
28	64.1	63	29.77	98	3.7
29	63.11	64	28.82	99	3.24
30	62.11	65	27.86	100	2.79
31	61.12	66	26.91	101	2.36
32	60.13	67	25.96	102	1.94
33	59.13	68	25.02	103	1.59
34	58.14	69	24.08	104	1.28
				105	1.02

Note: SEYLL, standard expected years of life lost (from Global Health Estimates Technical Paper WHO/HIS/HSI/GHE/2013.4).

Table S2. Spearman correlations between air pollutants and meteorological conditions in Ningbo, China, 2013–2016.

Variables	Ozone 8 h	$PM_{2.5}$	NO_2	SO_2	Temperature
Ozone 8 h (μg/m^3)	1.00				
$PM_{2.5}$ (μg/m^3)	−0.05*	1.00			
NO_2 (μg/m^3)	−0.21**	0.74**	1.00		
SO_2 (μg/m^3)	−0.15**	0.76**	0.70**	1.00	
Temperature (℃)	0.40**	−0.44**	−0.54**	−0.48**	1.00
Relative humidity (%)	−0.40**	−0.22**	−0.13**	−0.38**	0.14**

Note: *$p < 0.05$, **$p < 0.01$

Table S3. Changes in excess risk of hypertension mortality associated with 10 μg/m^3 increase in ozone exposure in the elderly in Ningbo, China, 2013–2016.

Variables	All （95% CI）	Spring-born （95% CI）	Summer-born （95% CI）	Autumn-born （95% CI）	Winter-born （95% CI）
**Single-pollutant model**	2.01(0.35, 3.67)	1.48(−2.25, 5.23)	1.30(−2.10, 4.72)	4.99(2.06, 7.92)	0.10(−2.74, 2.95) *
**Two-pollutant models**					
+$PM_{2.5}$	2.33(0.50, 4.17)	1.78(−2.36, 5.93)	1.78(−1.98, 5.54)	5.78(2.51, 9.06)	0.03(−3.16, 3.23) *
+NO_2	2.20(0.45, 3.95)	1.33(−2.60, 5.27)	1.46(−2.13, 5.07)	5.81(2.70, 8.93)	0.02(−3.01, 3.06) *
+SO_2	2.17(0.43, 3.92)	0.85(−3.05, 4.77)	1.38(−2.19, 4.96)	5.94(2.83, 9.05)	0.22(−2.80, 3.25) *

Changes were presented at 6-day moving average concentration (mv06). Values are shown as percentage. Note: *$p < 0.05$ indicated compared with autumn-born.
